# Maternal obesity disrupts the methionine cycle in baboon pregnancy

**DOI:** 10.14814/phy2.12564

**Published:** 2015-11-04

**Authors:** Peter W Nathanielsz, Jian Yan, Ralph Green, Mark Nijland, Joshua W Miller, Guoyao Wu, Thomas J McDonald, Marie A Caudill

**Affiliations:** 1 Center for Pregnancy and Newborn Research, Department OB/GYN, University of Texas Health Science Center San AntonioSan Antonio, Texas; 2 Texas Center for Pregnancy and Life Course Health, Southwest National Primate Research Institute, Texas Biomedical Research InstituteSan Antonio, Texas; 3 Division of Nutritional Sciences, Savage HallIthaca, New York; 4 Department of Medical Pathology and Laboratory Medicine, University of California Davis Medical CenterSacramento, California; 5 Department of Nutritional Sciences, Rutgers UniversityNew Brunswick, New Jersey; 6 Department of Animal Science and Center for Animal Biotechnology and Genomics, and Faculty of Nutrition, Texas A&M UniversityCollege Station, Texas

**Keywords:** Baboon, folate, maternal obesity, methionine cycle, vitamin B-12, one-carbon metabolism

## Abstract

Maternal intake of dietary methyl-micronutrients (e.g. folate, choline, betaine and vitamin B-12) during pregnancy is essential for normal maternal and fetal methionine metabolism, and is critical for important metabolic processes including those involved in developmental programming. Maternal obesity and nutrient excess during pregnancy influence developmental programming potentially predisposing adult offspring to a variety of chronic health problems. In the present study, we hypothesized that maternal obesity would dysregulate the maternal and fetal methionine cycle. To test this hypothesis, we developed a nulliparous baboon obesity model fed a high fat, high energy diet (HF-HED) prior to and during gestation, and examined methionine cycle biomarkers (e.g., circulating concentrations of homocysteine, methionine, choline, betaine, key amino acids, folate, and vitamin B-12). Animals were group housed allowing full physical activity and social interaction. Maternal prepregnancy percent body fat was 5% in controls and 19% in HF-HED mothers, while fetal weight was 16% lower in offspring of HF-HED mothers at term. Maternal and fetal homocysteine were higher, while maternal and fetal vitamin B-12 and betaine were lower in the HF-HED group. Elevations in circulating maternal folate were evident in the HF-HED group indicating impaired folate metabolism (methyl-trap) as a consequence of maternal vitamin B-12 depletion. Finally, fetal methionine, glycine, serine, and taurine were lower in the HF-HED fetuses. These data show that maternal obesity disturbs the methionine cycle in primate pregnancy, providing a mechanism for the epigenetic changes observed among obese pregnant women and suggesting diagnostic and therapeutic opportunities in human pregnancies complicated by obesity.

## Introduction

Nutritional imbalance and obesity during pregnancy predispose adult offspring to a variety of chronic health problems through a process known as developmental (or fetal) programming (Barker 1999; Armitage et al. 2004; Fernandez-Twinn and Ozanne 2010; Alfaradhi and Ozanne 2011; Sullivan et al. 2011; Shasa et al. 2015). Fetal adaptation to an abnormal nutritional environment is accompanied by changes in gene expression that are mediated (at least in part) by epigenetic modifications such as DNA and histone methylation (Saffery 2014). Epigenetic modification of the fetal genome can have profound effects on gene function, organ development, and metabolic processes throughout the lifespan of the offspring.

**Figure 1:**
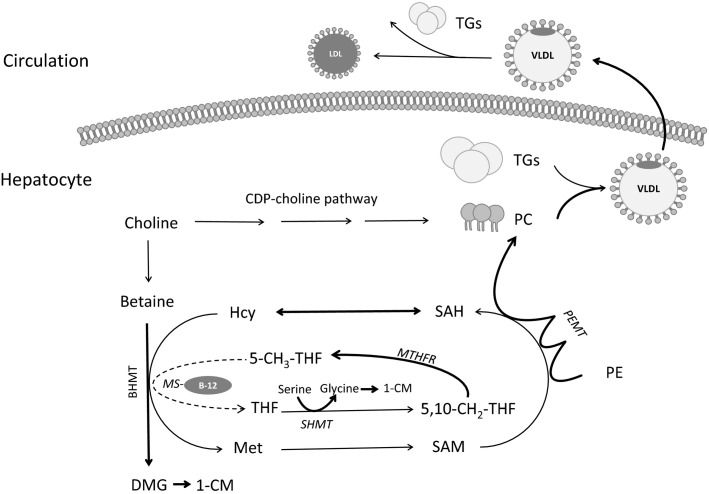
Simplified diagram of one-carbon metabolism (1-CM) including the methionine cycle, the roles of one-carbon nutrients, and links to lipid metabolism. Methionine (Met) can be synthesized from homocysteine (Hcy) in a reaction catalyzed by betaine-homocysteine methyltransferase (BHMT) using the choline metabolite betaine as the methyl donor. Alternatively, the remethylation of homocysteine to methionine can proceed via the folate and vitamin B-12 dependent methionine synthase (MS) reaction. Choline, betaine, folate, and vitamin B-12 have critical roles in lipid metabolism because they contribute to the production of phosphatidylcholine (PC) via the phosphatidylethanolamine-*N-*methyltransferase (PEMT) pathway, which is necessary for the formation of very-low-density-lipoproteins (VLDL) and the removal of triglycerides (TGs) from the liver. DMG, dimethylglycine; LDL, low density lipoprotein; MTHFR, 5,10-methylenetetrahydrofolate reductase; PE, phosphatidylethanolamine; SAM, S-adenosylmethionine; SAH, S-adenosylhomocysteine; SHMT, serine hydroxymethyltransferase; THF, tetrahydrofolate.

Within the one-carbon metabolic network, the methionine cycle (Fig.1) provides methyl groups from methionine and other dietary methyl-micronutrients (e.g. folate, vitamin B-12, choline, and betaine) to several important metabolic processes (Kalhan and Marczewski 2012) including those involved in developmental programming. While there are studies showing disturbances in the methionine cycle and gene specific DNA methylation as a result of maternal nutritional imbalances in rodent pregnancy (Lillycrop et al. 2010), few studies have been conducted in pregnant nonhuman primates (Blocker et al. 1989; Burbacher et al. 2004; McCurdy 2009). The need for data in primate pregnancy to translate to the human situation is evident from observed differences in the one-carbon and methionine cycle pathways between rodents and primates. For example, reduction of folic acid to tetrahydrofolate (THF; the active form of folate) by dihydrofolate reductase in the human liver occurs at less than 2% the rate observed in rat liver (Bailey and Ayling 2009). This lower rate of reduction in humans may result in the accumulation of dihydrofolate, which can compete with THF as a substrate for polyglutamyl synthase, resulting in diminished levels of the active folate polyglutamate cofactors required for one-carbon metabolism.

In the present study, a baboon obesity model was developed to investigate the effects of maternal obesity combined with a high fat, high energy diet (HF-HED) before and during pregnancy on maternal and fetal indicators of one-carbon metabolism and the methionine cycle. We hypothesized that several metabolic abnormalities would be detected including alterations in: (1) the maternal and fetal circulating profile of B-vitamins involved in the methionine cycle; (2) methionine cycle intermediates (e.g., homocysteine); and (3) amino acids that play a role in one-carbon metabolism (e.g., serine). Since the methionine cycle is involved in hepatic lipid metabolism and transport (Fig.1) (Caudill et al. 2012), we also measured biomarkers of fetal and maternal lipid status.

## Methods

### Subjects and diets

All animal procedures were approved by the Texas Biomedical Research Institute and University of Texas Health Science Center at San Antonio Institutional Animal Care and Use Committee, and conducted in AAALAC International approved facilities. Animals were grouped up to 16 animals per cage which allowed free movement and full social interaction. Healthy, nonpregnant female nulliparous baboons of similar weight, body dimensions, and age were randomly assigned to either a control diet (*n* = 22) or a combination of the control diet and a high fat-high energy diet (HF-FED) with free access to a sugar-containing drink (Kool Aid) (*n* = 5). The control group had continuous access to the Southwest National Regional Primate Research Center biscuits (Purina Monkey Diet and Monkey Diet Jumbo, Purina LabDiets, St Louis, MO) containing 12% energy from fat, 0.29% from glucose, 0.32% from fructose, and a metabolizable energy content of 3.07 kcal/g. The HF-HED group had continuous access to both the control diet and the HF-HED regimen containing 45% energy from fat, 4.6% from glucose, 5.6% from fructose, and a metabolizable energy content of 4.03 kcal/g as described previously (Maloyan et al. 2013). Both diets were made available to the HF-HED group because baboons ate more HF-HED when it was provided together with the control diet. HF-HED baboons were also given free and continuous access to a sugar containing Kool Aid drink. The control diet and HF-HED provided the same amount of vitamin B-12 (33 *μ*g/kg) and choline chloride (1200 ppm). However, folic acid was provided at levels of 2.2 ppm in the control diet, and 1.3 ppm in the HF-HED diet, while methionine comprised 0.31% of the ration in control diet and 0.33% in the HF-HED. The sugar-containing beverage (Kool Aid) contained no folate, choline, betaine, vitamin B-12, or amino acids.

Because all animals in the group cage had continuous access to the food, individual food intake was not assessed. Instead the daily intake per cage was measured and divided by the number of animals in the cage. Thus, food intake measurements are estimates per animal – a limitation inevitable in all studies using group housing to allow full activity and socialization. Females were maintained on either the control diet for 37 ± 0.3 months (mean ± SEM) or the combined control diet and HF-HED for 35 ± 0.8 months prior to breeding at 7.1 ± 0.1 (control) and 7.3 ± 0.1 (HF-HED; ns) years of age. Maternal morphometrics were performed at the time of group assembly and again after 3, 9, and 18 months; Dual Beam X-ray absorptiometry (DEXA) was performed at the time of group assembly and again after 6, 12, and 18 months; and measures of serum LDL-cholesterol and triglycerides in venous blood samples were performed at the time of group assembly and again after 3 months. No additional assessments were conducted after 18 months of the prepregnancy period.

**Breeding and animal management** has been described in detail (Schlabritz-Loutsevitch et al. 2004). Control and HF-HED baboons were housed in groups in separate cages to prevent control females from consuming components of the HF-HED. All animals remained on their prepregnancy diet throughout pregnancy. Fetuses were delivered at 0.9 gestation (G; Term 184 days) via cesarean section under general anesthesia as previously described (Cox et al. 2006). Prior to removal, the fetus was exsanguinated while still under general anesthesia, a method approved by the American Veterinary Medical Association, and checked for response to a footpad pinch with a forcep. Morphometric measurements were made on mothers at baseline and delivery, and on the fetus at delivery. Blood samples for metabolite measurements were taken from the maternal uterine vein and fetal umbilical vein at C-Section, and processed to provide serum and plasma which were stored at −80°C.

### Metabolite measurements

*Amino acids*: Serum amino acids were determined by HPLC with precolumn derivatization with o-phthaldialdehyde (Wu and Knabe 1994). All amino acids in the samples were quantified on the basis of authentic standards (Sigma Chemicals, St. Louis, MO) using Millenium-32 Software (Waters, Milford, MA).

*Maternal and fetal serum vitamin B12, folate and homocysteine* were measured on an Immulite 1000.

*Holotranscobalamin***:** Plasma holotranscobalamin was measured by the monoclonal antibody capture assay (Ulleland et al. 2002).

*Lipid measures:* Serum triglycerides and LDL-cholesterol were measured enzymatically (Rainwater et al. 2002a, 2007). Concentrations of serum apolipoproteins A1 (ApoA1) and B (ApoB) were quantified immunoturbidometrically (Kammerer et al. 2002; Rainwater et al. 2002b).

*Free choline, betaine, and dimethylglycine (DMG)* were quantified in plasma using liquid chromatography-tandem mass spectrometry (LC-MS/MS) (Holm et al. 2003) with modifications based on the instrumentation (Yan et al. 2011). The metabolites and internal standards (d_9_-choline, d_9_-betaine, and d_3_-dimethylglycine) were detected as positive ions by tandem mass spectroscopy in the multiple-reaction monitoring mode, using molecular transitions of *m/z* 104→60 (choline), *m/z* 113→69 (d_9_-choline), *m/z* 118→59 (betaine), *m/z* 127→68 (d_9_-betaine), *m/z* 104→58 (DMG), and *m/z* 107→61 (d_3_-DMG). For all three metabolites, the within- and between day imprecision (CVs) were <6%.

### Statistical analysis

Analysis was conducted by either Student’s *t*-test or repeated measures ANOVA; Bonferroni multiple comparison test was used for mean group separation. Differences were considered statistically significant at *P *≤* *0.05, while 0.05 < *P *≤* *0.10 was indicative of borderline significance. Since the sex distribution of the five HF-HED fetuses was three females and two males, no attempt was made to analyze the data according to fetal sex. Data are presented as means ± SEM.

## Results

### Food intake and weight changes during the prepregnancy period

**Figure 2:**
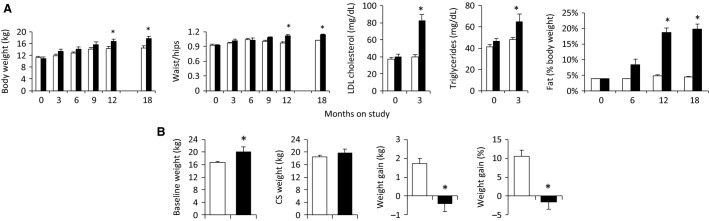
(A) Maternal prepregnancy anthropometrics over the first 18 months on the high fat–high energy diet (HF-HED), and circulating lipids at 0 and 3 months in control (CTR, open, *n* = 7) and HF-HED (filled, *n* = 5) animals. (B) Baseline body weight before pregnancy, weight at C-section (CS), and weight gained during pregnancy in CTR (open, *n* = 22) and HF-HED animals (filled, *n* = 5). Data are mean ± SEM; *P < 0.05 versus CTR.

Total weight gain in the first year prior to pregnancy was significantly higher (*P* < 0.001) in the HF-HED group (5.8 kg) as compared to the control group (3.1 kg) (Fig.2A). During the prepregnancy period control baboons ate 22 g/kg body weight per day (68 kcal/kg/day) of the control diet while HF-HED mothers ate 13.6 g/kg per day of the control diet (42 kcal/kg/day) and 3.8 g/kg per day of the HF-HED diet (15 kcal/kg/day) for a total calorie intake of 57 kcal/kg/day, which is numerically lower than the control group. However, when expressed as an absolute value, daily caloric intake from the diets was similar among the groups (∼1000 kcal/day), and was further increased in the HF-HED group via their consumption of the high calorie Kool-Aid. The HF-HED group also exhibited higher waist-hip circumferences, higher LDL-cholesterol and triglycerides, and a greater percentage body fat during the prepregnancy period (*P* < 0.05, Fig.2A).

### Weight changes during pregnancy

Baseline body weight at pregnancy was higher (*P* < 0.05) in HF-HED versus control (Fig.2B). During pregnancy, HF-HED mothers failed to gain weight, while the control mothers gained 1.8 kg (Fig.2B). Otherwise pregnancies in both groups were uneventful.

### Fetal morphometrics at 0.9 gestation (G)

Table1 presents the morphometric characteristics of the placenta and fetuses of control and HF-HED mothers at Cesarean section. Although there were no differences in placental weight or volume, placental efficiency expressed as weight of fetal mass supported per unit placental mass, was lower in HF-HED pregnancies (Table1). Fetal body weight was 16.3% lower (*P* < 0.05) in HF-HED pregnancies (vs. control) and was also lower (*P* < 0.05) than control as a percent of maternal weight. The only fetal organ difference to approach significance in absolute weight between the two groups was lower fetal liver weight (*P* = 0.07) in the HF-HED group (vs. control). When fetal organ weights were expressed as a percentage of fetal weight, fetal brain and thymus weights were higher (*P* < 0.05) in HF-HED pregnancies suggesting some degree of tissue sparing for these organs.

**Table 1:** Placental and fetal morphometric characteristics at cesarean section (0.9 gestation) in control (*n* = 20–22) and HF-HED (*n* = 5) group*

	Control	HF-HED
Placenta Measures		
Weight (g)	210 ± 9	214 ± 9
Efficiency (g fetus/g placenta)	3.9 ± 0.1	3.2 ± 0.2†
Volume (cm^3^)	159 ± 18	111 ± 26
Fetal Measures		
Body weight (g)	806 ± 24	675 ± 36†
Length (cm)	38 ± 0.6	37 ± 1
BMI (kg/m^2^)	5.7 ± 0.1	5.1 ± 0.4‡
Ponderal index (kg/m^3^)	15 ± 0.5	14 ± 1
Brain (g)	82 ± 2	80 ± 2
Heart (g)	4.9 ± 0.2	4.2 ± 0.2
Kidneys (g)	4.5 ± 0.2	4.0 ± 0.2
Liver (g)	24 ± 0.9	20 ± 1.2‡
Lung (g)	17 ± 0.6	17 ± 1.5
Pancreas (g)	0.56 ± 0.06	0.44 ± 0.05
Thymus (g)	3.4 ± 0.20	3.4 ± 0.46
Fetal measures as percent fetal weight		
Placenta	26 ± 0.8	32 ± 2.5†
Brain	11 ± 0.3	12 ± 0.6†
Heart	0.61 ± 0.02	0.63 ± 0.04
Kidneys	0.56 ± 0.02	0.59 ± 0.03
Liver	3.0 ± 0.06	3.0 ± 0.125
Lungs	2.2 ± 0.07	2.4 ± 0.12‡
Pancreas	0.07 ± 0.01	0.07 ± 0.01
Thymus	0.42 ± 0.02	0.52 ± 0.05†
Fetal measures as percent maternal weight		
Fetal weight	4.3 ± 0.1	3.5 ± 0.2†
Placenta	1.13 ± 0.04	1.09 ± 0.05

* Data are mean ± SEM.

† *P* < 0.05 versus control.

‡ 0.05 < *P* < 0.10 versus control.

### Maternal and fetal methionine cycle metabolites

**Figure 3:**
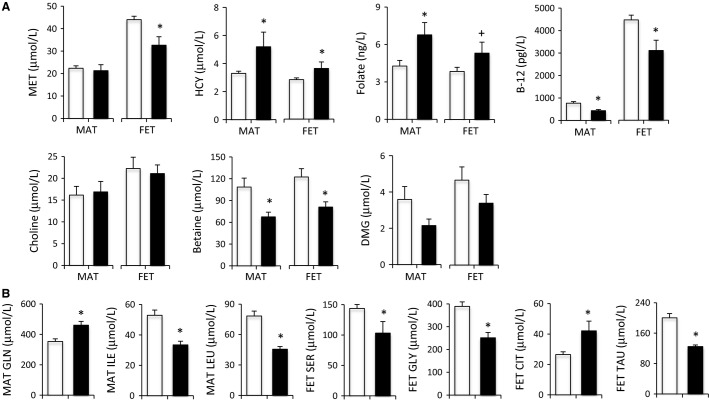
(A) Maternal (MAT) and fetal (FET) circulating concentrations of methionine cycle metabolites and biomarkers in control (CTR, open) and high fat-high energy diet (HF-HED, closed) at 0.9 G (*n* = 11–22 CTR MAT; *n* = 5 HF-HED MAT; *n* = 11–21 CTR FET; *n* = 5 HF-HED FET). (B) Amino acids in CTR (open, *n* = 21–22) and HF-HED (filled, *n* = 5) MAT and FET blood at 0.9 G. Data are mean ± SEM; *P < 0.05 versus CTR; ^†^0.05 < *P* < 0.10 versus CTR. CTT, citrulline; DMG, dimethylglycine; GLN, glutamine; GLY, glycine; HCY, homocysteine; ILE, isoleucine; LEU, leucine; MET, methionine; SER, serine; TAU, taurine; and TC, holotranscobalamin.

Methionine cycle metabolites in the maternal and fetal baboon at Cesarean section are shown in Figure3A. While methionine was unchanged in maternal plasma, concentrations were 28 percent lower in fetuses of the HF-HED mothers than controls (*P* < 0.05) (Fig.3A). Homocysteine was 36% higher in maternal blood and 28% higher in fetal blood in the HF-HED group (vs. control) (*P* < 0.05). Vitamin B-12 was lower (*P* < 0.05) in mothers (−44%) and fetuses (−31%) in the HF-HED group (vs. control), while lower, but nonsignificant levels of holotranscobalamin were observed in maternal blood (−36%) and in fetal blood (−21%) in the HF-HED group. In contrast, folate was 59% higher (*P* < 0.05) in maternal blood in the HF-HED group (vs. control) and tended (*P* < 0.10) to be higher (+38%) in fetal blood. Betaine, which is derived from choline and serves as an alternate to folate as a source of methyl groups for conversion of homocysteine to methionine, was lower in both the maternal (−39%; *P* < 0.02) and fetal (−36%, *P* < 0.02) blood in the HF-HED group (vs. control). Dimethylglycine, the metabolite produced when betaine is used as a methyl donor and source of one-carbon units for folate-mediated one-carbon metabolism, tended to be lower (−45%) in maternal blood (*P* < 0.1) of the HF-HED group (vs. control); fetal blood also exhibited lower dimethylglycine but statistical significance was not achieved (Fig.3). Circulating choline was unchanged by the HF-HED diet in either mother or fetus.

### Maternal and fetal amino acid concentrations

HF-HED mothers had higher concentrations of plasma glutamine and lower concentrations of leucine and isoleucine, while fetuses of HF-HED mothers (vs. control) had lower plasma concentrations of glycine, serine, and taurine and higher citrulline (all *P* < 0.05; Fig.3B). The fetal:maternal ratio was lower (*P* < 0.03) in the HF-HED pregnancies (vs. control) for the following five amino acids: serine (1.46 ± 0.23 vs. 2.19 ± 0.10), glycine (1.06 ± 0.08 vs. 1.74 ± 0.08), taurine (1.10 ± 0.12 vs. 1.44 ± 0.05), methionine (1.56 ± 0.12 vs. 2.08 ± 0.07) and phenylalanine (1.09 ± 0.10 vs. 1.47 ± 0.07). The ratio was significantly higher (*P* < 0.01) in the HF-HED pregnancies (vs. control) for citrulline (3.76 ± 0.84 vs. 2.09 ± 0.09), isoleucine (2.25 ± 0.22 vs. 1.51 ± 0.08), and leucine (2.20 ± 0.16 vs. 1.40 ± 0.08). No other differences in amino acids were detected between the HF-HED and control pregnancies.

### Maternal and fetal lipid metabolism

**Figure 4:**
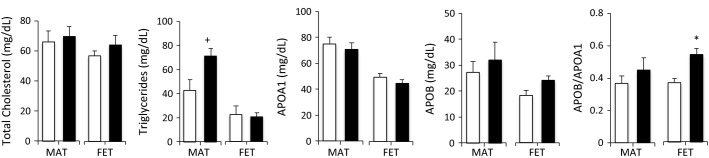
Lipid parameters in baboon pregnancy at 0.9 G in control (open, *n* = 8–11) and HF-HED (filled, *n* = 5) maternal (MAT) and fetal (FET) blood. Data are mean ± SEM; *P < 0.05 versus CTR; ^†^0.05 < *P* < 0.10 versus CTR. APOA1, Apolipoprotein A1; APOB, Apolipoprotein B.

Maternal triglycerides at C-section were 67% higher (*P* = 0.06) in the HF-HED group (vs. control). Although fetal triglycerides were similar among groups, fetal APOB tended to be higher (+32%) in the fetuses of HF-HED mothers (*P* = 0.08) and the APOB/APOA1 ratio was significantly higher (*P* < 0.05) (Fig.** **4).

## Discussion

Obesity and excessive nutrient intake are an ever-growing health concern in our over-consuming society. Abundant data indicate that maternal obesity in pregnancy leads to adverse developmental programming of offspring outcomes in rodents (Samuelsson et al. 2008; Alfaradhi et al. 2014), sheep (Sinclair et al. 2007; Long et al. 2012; Shasa et al. 2015), nonhuman primates (Aagaard-Tillery et al. 2008; Grayson et al. 2010; Sullivan et al. 2010; Grant et al. 2011; Suter et al. 2011; Maloyan et al. 2013), and humans (Gillman et al. 2008). However, despite the importance of one-carbon metabolism in a wide array of cellular and molecular processes fundamental to development, little is known regarding the effects of maternal obesity on the methionine cycle in mother and fetus, and there are no studies to provide guidance in this epidemic situation of obese pregnancies.

We developed a pregnant baboon model of obesity by feeding a HF-HED before and during pregnancy and comparing to controls consuming the normal primate center control diet. Nulliparous female baboons of the HF-HED (vs. control) group exhibited higher body fat percentages, higher waist-hip circumferences, and dyslipidemia (elevated LDL-C and triglycerides). Borderline higher circulating triglycerides were also detected in the HF-HED (vs. control) female baboons at delivery which is consistent with an obesogenic phenotype. The lack of significant weight gain and smaller fetuses among the baboon mothers in the HF-HED (vs. control) group has parallels in human obesity. Obese women gain less weight than their nonobese counterparts during pregnancy (Faucher and Barger 2015) and small-for-gestational-age births, which are more likely to occur when the mother fails to gain weight during pregnancy, can affect up to 14 percent of obese pregnancies (Faucher and Barger 2015).

### Maternal obesity alters circulating concentrations of methyl-nutrients

Maternal and fetal blood concentrations of vitamin B-12 were depleted in the HF-HED group as compared with the control group. In contrast, blood folate concentrations were elevated in maternal blood and tended to be elevated in fetal blood. A possible explanation for the rise in folate is impaired vitamin B-12 dependent methionine synthase activity, with subsequent cellular accumulation of 5-methyl-THF (i.e., metabolic trapping of folate; methyl trap) (Caudill et al. 2012). Methionine synthase catalyzes the transfer of a methyl group from 5-methyl-THF to homocysteine generating methionine and THF. Consequently, in vitamin B-12 deficiency, cellular and circulating levels of homocysteine and 5-methyl-THF will rise. The vitamin B-12 deficiency that led to the metabolic trapping of folate in the HF-HED group may be related to the caloric/lipid load (McCurdy 2009) and increased use of vitamin B-12 as a lipotrope. By providing methyl groups for de novo synthesis of phosphatidylcholine, lipotropes facilitate VLDL biosynthesis and hepatic lipid export (Lamaziere et al. 2012) (Fig.1). In contrast, although absolute vitamin B-12 intake may have been slightly lower among the HF-HED (vs. control) group, this modestly lower intake would not be expected to cause the functional B-12 deficiency observed in the HF-HED moms.

Maternal and fetal blood concentrations of the choline metabolite, betaine, were significantly reduced in HF-HED compared with controls, while dimethylglycine tended to be lower in maternal blood of the HF-HED group. Betaine is used as a methyl donor by betaine-homocysteine methlytransferase (BHMT), an alternative to methionine synthase for conversion of homocysteine to methionine (Fig.1). Like vitamin B-12, betaine is a lipotrope and its use would be augmented under conditions of a lipid load. Similarly, although dimethylglycine is a product of the BHMT reaction, it is also a source of one-carbon units (e.g., methyl groups) which can be used in folate mediated one-carbon metabolism including the methionine cycle.

### Maternal obesity alters one-carbon amino acid concentrations in fetal blood

A novel and important finding from the present study is that plasma methionine, serine, glycine, and taurine concentrations (amino acids involved in one-carbon metabolism and related pathways) were unchanged in the maternal circulation, but decreased in fetal plasma of HF-HED pregnant baboons resulting in lower fetal to maternal ratios for these amino acids. This finding may indicate placental dysfunction in relation to the activity of placental transporters for small and neutral amino acids (System ASC and SNAT) as well as taurine (System Beta). For example, placental microvilli preparations from obese pregnant women (BMI 32) exhibited reduced in vitro placental SNAT activity and SNAT4 expression compared to their lean counterparts (BMI 22) (Farley et al. 2010). The reduced placental efficiency (which has also been observed in obese human pregnancies; Wallace et al. 2012) and lower fetal weights of the HF-HED mothers are also consistent with impaired amino acid transport.

In contrast with the amino acids associated with one-carbon metabolism, fetal to maternal ratios for citrulline, leucine, and isoleucine were higher in the fetuses of the HF-HED mothers than controls, suggesting either up-regulation of transporters for branched-chain amino acids (e.g., System L) and citrulline (e.g., System N) or reduced catabolism of these amino acids in fetal tissue.

## Conclusions

In our baboon nonhuman primates, maternal obesity (even without weight gain during pregnancy) disrupted the methionine cycle and reduced several one-carbon nutrients particularly in the fetal compartment. These data provide a mechanism for the epigenetic changes observed in maternal obesity, but additional studies with genomic and epigenomic endpoints are required. The reduction of one-carbon nutrients in maternal obesity suggests that nutritional strategies aimed at achieving optimal concentrations of these key components of the methionine cycle (e.g. vitamin B-12 supplementation) may be warranted.
